# Biofilm elimination from infected root canals using four different single files

**DOI:** 10.1186/s12903-022-02690-5

**Published:** 2022-12-31

**Authors:** Sarah A. Hamed, Sarah Shabayek, Hayam Y. Hassan

**Affiliations:** 1 Endodontic Department, Faculty of Dentistry, Suez Canal University, Ismailia, Egypt; 2 Microbiology and Immunology Department, Faculty of Pharmacy, Suez Canal University, Ismailia, Egypt

**Keywords:** *Enterococcus faecalis* biofilm, XP-endo shaper, Hyflex DEM, One curve

## Abstract

**Introduction:**

*Enterococcus faecalis* (*E. faecalis*) is the most commonly isolated bacterium from infected root canals. It is found in the form of a biofilm, which makes it more resistant to antimicrobials, and requires optimal chemomechanical strategies to maximize root canal disinfection.

**Aim:**

To evaluate the efficacy of 4 different endodontic file systems against *E. faecalis* biofilm growth in root canals using colony-forming units per milliliter (CFU/mL) and scanning electron microscope (SEM).

**Methods:**

Eighty-five extracted human mandibular premolars with straight root canals and apical diameters not larger than the #15 K-file were randomly selected. After performing a pilot study (n = 15) to determine the ideal incubation period for *E. faecalis* biofilm development, sixty-five root canals were infected with *E. faecalis*, incubated for 3 weeks, and then mechanically prepared using one of four single files (XP-endo Shaper, Hyflex EDM, One Curve, and Fanta. AFTM F One) (n = 15). Five infected root canals were excluded for the positive control. Five non-contaminated root canals were included for the negative control. Samples were collected using sterile paper points pre- and post-instrumentation to determine the bacterial load (CFU/mL). Root canals from each group were topographically evaluated at the coronal, middle, and apical segments using scanning electron microscope (SEM). Bacterial reduction data were estimated and statistically analyzed by Kruskal–Wallis and Mann–Whitney U tests (post hoc test) (*P* ≤ .05).

**Results:**

XP-endo Shaper, Hyflex DEM, and One Curve significantly could eradicate *E. faecalis* biofilms in infected root canals with no significant difference among them compared to Fanta. AF™ F One.

**Conclusion:**

None of the systems were capable of completely eliminating biofilms. XP-endo Shaper, Hyflex EDM, and One Curve mechanically eliminated *E. faecalis* biofilms compared to Fanta. AF™ F One from infected root canals.

## Introduction

Endodontic therapy aims to completely eradicate microorganisms and their toxins produced in the root canal space [1]. However, *E. faecalis* is frequently isolated from persistent periapical lesions [2, 3] that often presents as biofilms [4]. *Enterococcus* *faecalis* inside dentinal tubules can survive away from intracanal medicaments such as calcium hydroxide for longer than 10 days if high pH cannot be maintained [5, 6].

Mechanical enlargement of the root canal allows irrigants to reach the entire root canal system, providing further debridement through flushing and antibacterial properties [7]. However, complete disinfection is not feasible because of the difficulty of the root canal system, which includes the dentinal tubules, isthmus, fins, and accessory canals which act as shelters to protect bacteria and their biofilms [8, 9]. Therefore, new chemomechanical strategies have been developed and studied to maximize root canal disinfection before obturation, particularly through experiments using an *E. faecalis* biofilm model.

Further, several techniques and materials have been invented, including root canal preparation using a single-file system that offers both time and cost savings compared to full sequential rotary systems [10]. Single-files have been demonstrated to be as effective as [11, 12] or even better than multiple file systems [13].

Recently, a snake-shaped file called XP-endo Shaper (XPS) (FKG Dentaire, La Chaux-de-Fonds, Switzerland) was made from a unique Max wire NiTi alloy. It has an initial taper of 0.01 in its martensite phase, when warmed at the body temperature inside the root canal it expands to a taper of at least 0.04 in its austenite phase becoming more serpentine. XPS has a booster tip with six cutting edges that respect the canal while cutting at each pass [14]. XPS has remarkable flexibility, fatigue resistance, super elasticity, and expansion or contraction according to the canal morphology [15].

Hyflex EDM file (HEDM) (Coltene/Whaledent AG, Altstatten, Switzerland) was made from CM wire using an electrical discharge machine (EDM), which hardens the surface of the file, increased fracture resistance and cutting efficiency. HEDM has 0.25 mm apical diameter, with regressive taper [16].

One Curve file [OC] [Micro-MEGA, Besancon, Cedex, France] was produced from C-Wire, which has controlled memory, making the file hyperflexible with increased cyclic fatigue resistance. It has a variable cross-section operated with continuous rotation that improve cutting efficiency and provide centered preparation preventing the sucking effect [17].

Fanta. AF™ F One (FO) (Shanghai Fanta Dental Material Co., China) is a recently introduced single-file system produced using the AF-R wire technique. As claimed by the manufacturer, it has an inactive tip and a unique flat-sided surface design, providing room for irrigant solutions during mechanical preparation, less stress on the file, and more flexibility without compromising strength [18].

This study evaluated the in vitro efficacy of the following single-file systems: XPS, HEDM, OC, and FO against *E.* *faecalis* biofilms from infected root canals. The null hypotheses suggested no differences between single-file systems used to eradicate *E. faecalis* biofilms from infected root canals.

## Methods

### Sample collection

This in vitro double-blind study was conducted on eighty-five unidentified extracted straight single-canaled mandibular premolars with apical diameters not larger than the #15 K file, extracted for orthodontic reasons and periodontal diseases. The teeth were extracted at the oral surgery and maxillofacial department in the Faculty of Dentistry, Suez Canal University. Prior to the start of the extraction, each patient signed a written informed consent form.

Teeth with curvatures, cracks, root caries, resorptive defects, calcifications, or teeth with endodontic treatment were excluded. The teeth were selected after obtaining periapical radiographs from both mesiodistal and buccolingual views. After cleaning the calculus and soft debris, the teeth were soaked for 1 h in 0.2% sodium azide for disinfection and stored in saline until use.

Under magnification of the dental loupes (Univet, Rezzato (BS), Italy), the tooth length was standardized to 17 mm from the apex by using a diamond disc (Mani, Tochigi, Japan). Modified access cavities were created for all roots. Apical patency was checked by passing K-file #15 (Mani, Japan). The working length was adjusted at 16 mm. K-files #10 and #15 were used to create the glide paths. A dose of 25 kGy gamma radiation was used to sterilize the teeth for 6 h [19]. All apical foramina were closed with epoxy resin and the roots were covered with two layers of nail varnish [20]. Five teeth were randomly selected and placed in Eppendorf tubes containing sterile nutrient brain heart infusion (BHI) broth as a negative control.

### Bacterial preparation

The inoculum was prepared by adding 24 h isolated colonies of a pure culture of *E. faecalis* strain (ATCC29212) to 15 mL of brain heart infusion (BHI) broth (Biolife Italiana S. r. I.; Viale Monza, Milan, Italy), and incubated at 37 °C for 24 h under aerobic conditions. The optical density of the bacterial suspension was adjusted until its turbidity recorded 0.5, on McFarland scale, matching to 1.5 × 10^8^ colony forming units per milliliter (CFU/mL).

### Pilot study

A pilot study was arranged to determine the optimal incubation period for *E. faecalis* biofilm formation. Under a laminar flow hood, the root canals of 15 randomly selected teeth were completely filled with 10 µL of 24 h *E. faecalis* suspension and incubated at 37 °C in 100% relative humidity for 5 weeks. Bacterial suspensions (10 µL) were added daily to maintain the culture viability [21]. *E. faecalis* biofilm formation was also assessed at five different time intervals (1, 2, 3, 4, and 5 weeks), three teeth at each one. The teeth were fixed by immersion in modified Karnovsky solution [22] and left overnight. They were split longitudinally and processed for scanning electron microscope [SEM] examination at 3000X magnification.

### Experimental study

Sample size was calculated using (G* Power) computerized software guided by the results of a previous study [23], yielding a minimum of 40 samples (10 samples/group). The sample size was increased to (15 per group) for teeth that may have been lost during the experiment, (effect size = 0.766, Pooled SD = 1.48, Alpha (α) = 0.05and 4. Power (β) = 0.99).

The pilot study proved that 3 weeks was the ideal incubation period for *E. faecalis* biofilm development. Under aseptic conditions, sixty-five teeth were inoculated with a previously prepared bacterial suspension for 24 h, as mentioned previously, and incubated for 3 weeks with daily addition of 24 h bacterial suspension. Eight teeth had cracks that were discarded, and the remaining teeth were coded and equally divided into 4 experimental groups. After incubation, the root canals were filled with 1 mL sterile saline and placed in sterile Eppendorf tubes. Before instrumentation, bacterial samples (S1) were collected using three sterilized #20 paper points. Each paper point was placed inside the root canal for 1 min, transferred to the corresponding Eppendorf tube, then vortexed for 30 min [24]. Suspensions were prepared through serial dilutions of (10 ^−2^, 10^−4^, and 10^−5^) and 0.1 mL aliquots of several dilutions were streaked onto M-Enterococcus agar plates that were incubated at 37 °C with 5% Co_2_ for 48 h. Colonies were counted by calculating the number of colony-forming units per milliliter (CFU/mL).

### Samples preparation

Five roots containing bacteria were used as positive controls to assess the bacterial viability throughout the experiment. The remaining fifty-two roots were randomly divided into four groups (n = 13) corresponding to the file used for instrumentation. Root canal instrumentation was accomplished according to the manufacturer’s instructions using rotary motion generated by a torque-controlled electric motor (seongseo ro, Daegu, Korea) with a 16:1 gear reduction contra-angle handpiece. During instrumentation, the samples were immersed in a warm water bath at 37 ± 1 °C [25, 26].

In group A, XPS (#30/0.04) was performed at 800 rpm and 1 Ncm. It was applied with gentle and slow up-and-down strokes reaching the working length. In group B, HEDM (#25/ ~) was adjusted at 500 rpm and 2.5 Ncm in up and down movement. In group C, OC (#25/0.06) was operated at 300 rpm and torque 2.5 Ncm in pecking motion till reaching the working length. In group FO (#25/0.06) was operated at 500 rpm and torque 2.6 Ncm in up and down movement. Each file was discarded after shaping four canals.

After each of the four up-and-down movements, all root canals were irrigated with 3 mL 2.5% sodium hypochlorite for 1 min by a 30-gauge irrigation needle. Finally, 5 mL of 17% ethylenediaminetetraacetic acid (EDTA) were used as a final rinse for 3 min. Next, 2 mL distilled water was used.

After instrumentation, the second bacterial samples (S2) were collected from the root canals using three sterilized #20 paper points, as described previously [27]. Aliquots (0.1 mL aliquots) were cultured on M-Enterococcus agar plates and incubated at 37 °C for 48 h. Colonies were counted and transferred to actual counts according to previously recorded dilution factors. Roots from each group were fixed, split longitudinally using a chisel and mallet, and dehydrated by immersion in ethanol (50, 80, 90, 96, and 100%). Each root canal lumen was topographically evaluated in the coronal, middle, and apical segments using SEM [Jeol JSM-6510L.V, Jeol, Tokyo, Japan] at 3000X, 6000X, and 12000X magnifications.

The data were statistically analyzed using the Kruskal–Wallis test, followed by the Mann–Whitney U test, to compare the efficiency of the used files to reduce the bacterial count (*P* < .05). The data was analyzed using SPSS (statistical package for social science, version 25).

## Results

### The pilot study

**Fig. 1:**
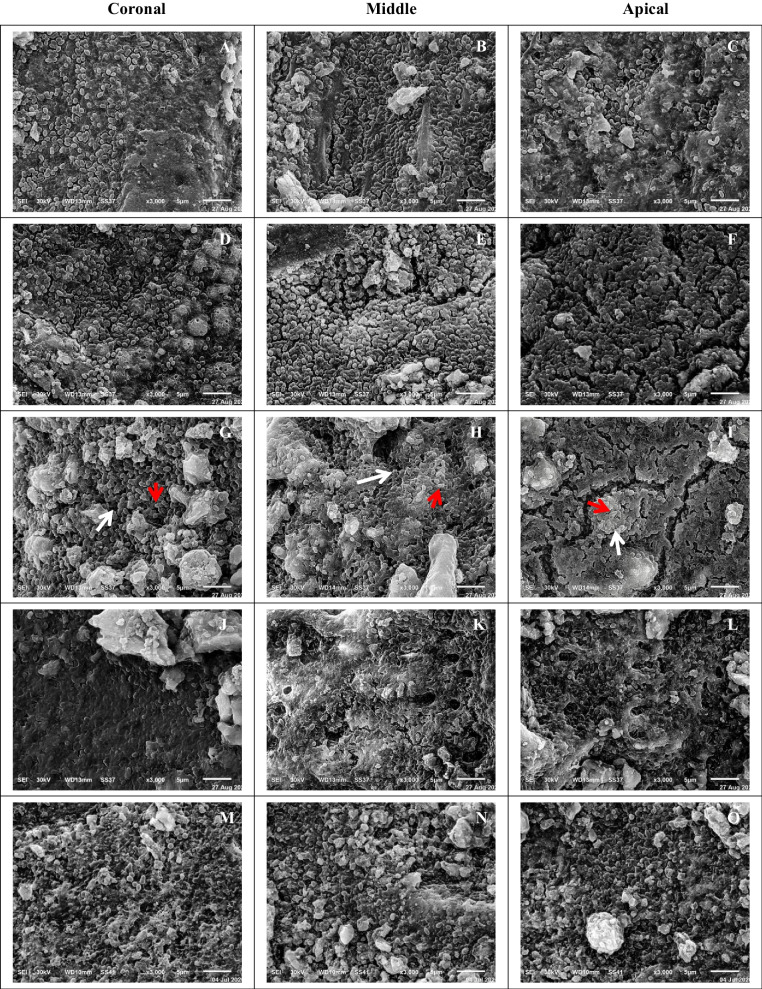
SEM views showing stages of *E. faecalis* biofilm formation and maturation onto root canal dentin at 3000X. **A**–**C** after 1 week. **D**–**F** after 2 weeks. **G**–**I** after 3 weeks, showing mature biofilm (white arrows) with actively dividing bacteria (red arrows). **J**–**L** after 4 weeks. **M**–**O** after 5 weeks

In this study, it was proven that the ideal incubation time for *E. faecalis* biofilm development and maturation is 3 weeks, as shown in Fig. 1.

### The experimental study

**Fig. 2:**
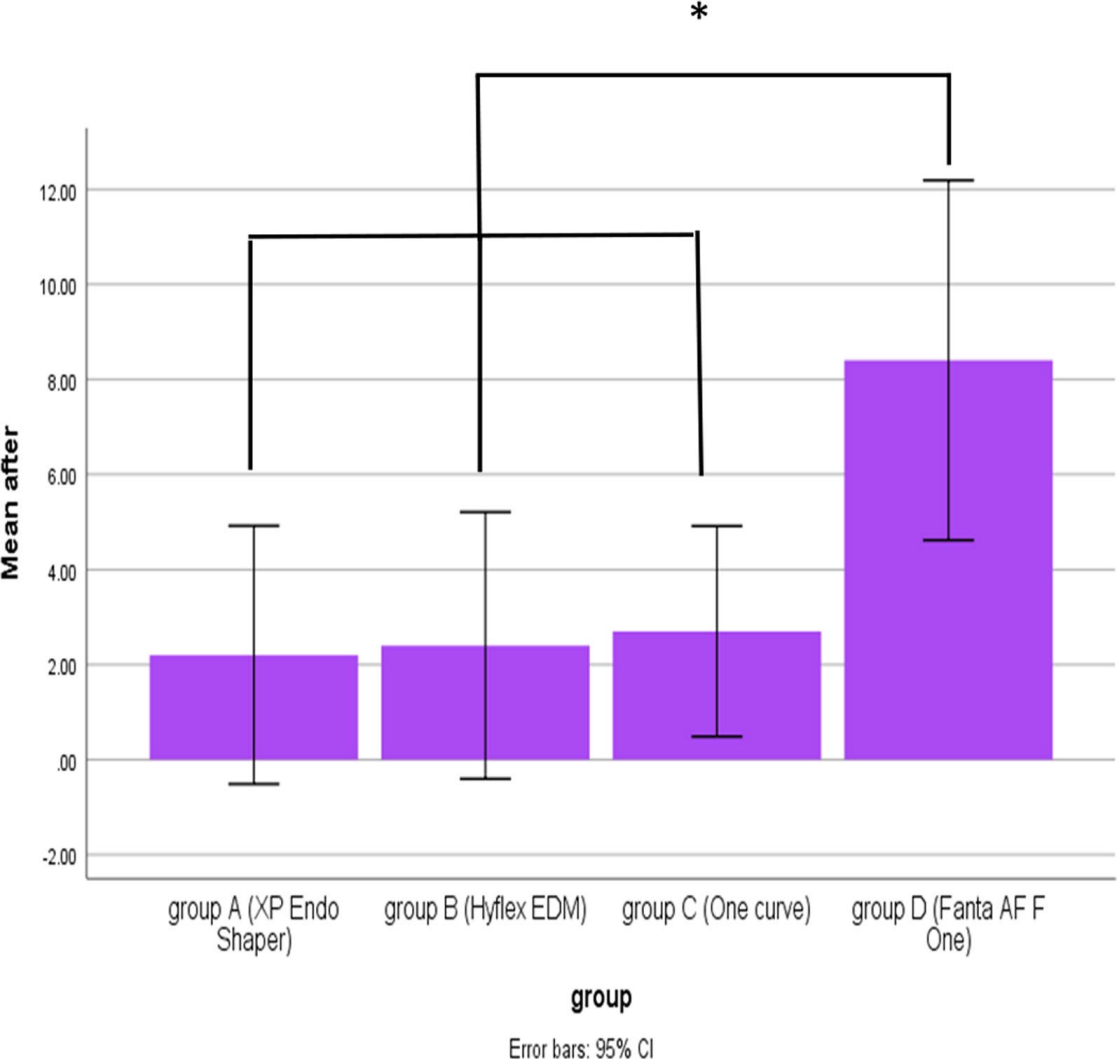
Comparison of mean bacterial count (× 105 CFU/ml) between groups after instrumentation using the four tested file systems

No bacterial growth was detected in the negative control group. Therefore, the scanned samples of the negative controls showed that the root canal dentin surface was covered by debris without bacteria or biofilms. The positive control group exhibited bacterial growth throughout the experiment. A comparison of the mean CFU count (× 10^5^ CFU/mL) between the groups before and after instrumentation using the four tested files were shown in Fig. 2

**Fig. 3:**
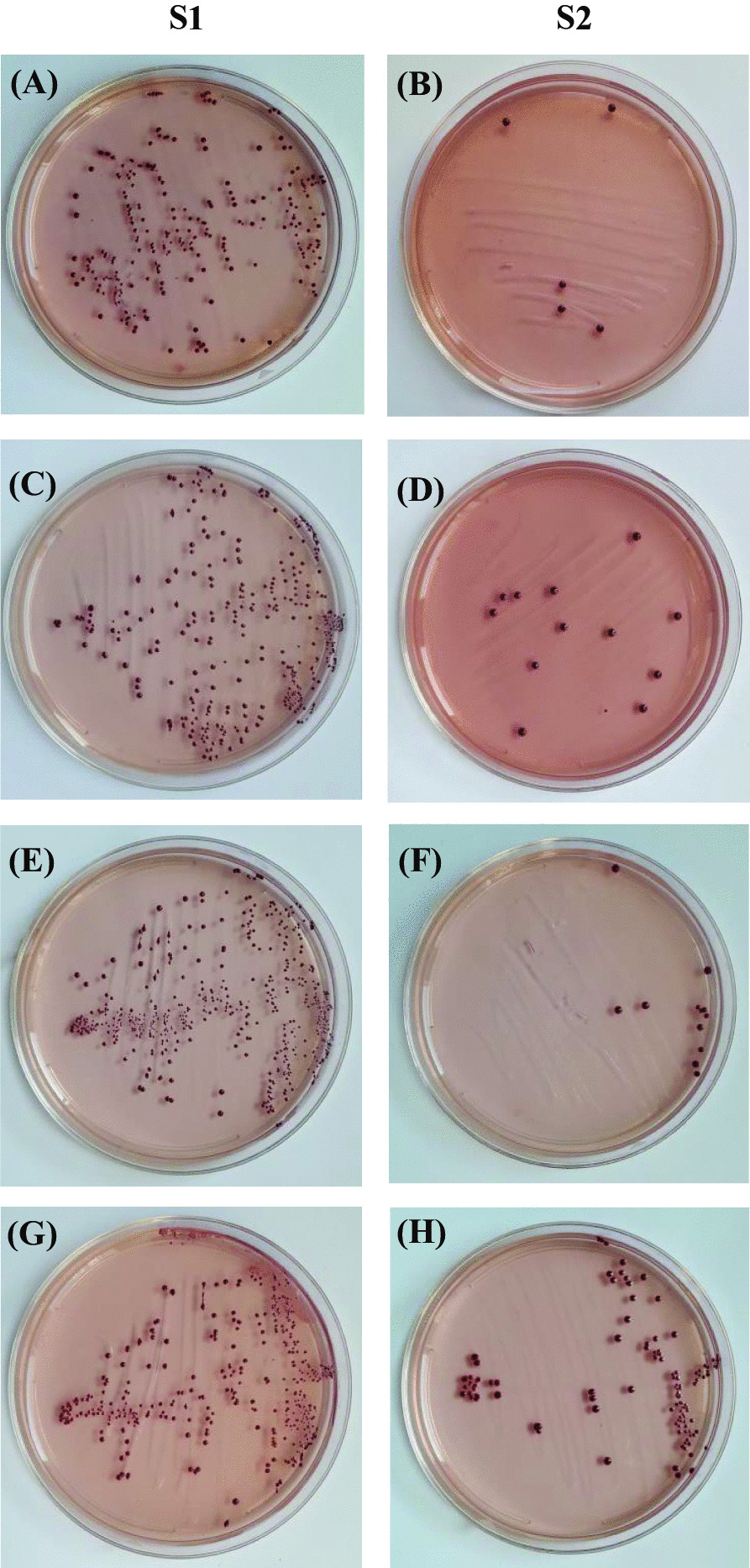
Photographs showing comparison between changes in the bacterial count before (S1) and after instrumentation (S2). **A**, **B** for XP-endo Shaper. **C**, **D** for Hyflex EDM. **E**, **F** for One Curve. **G**, **H** for Fanta. AFTM F One

**Fig. 4:**
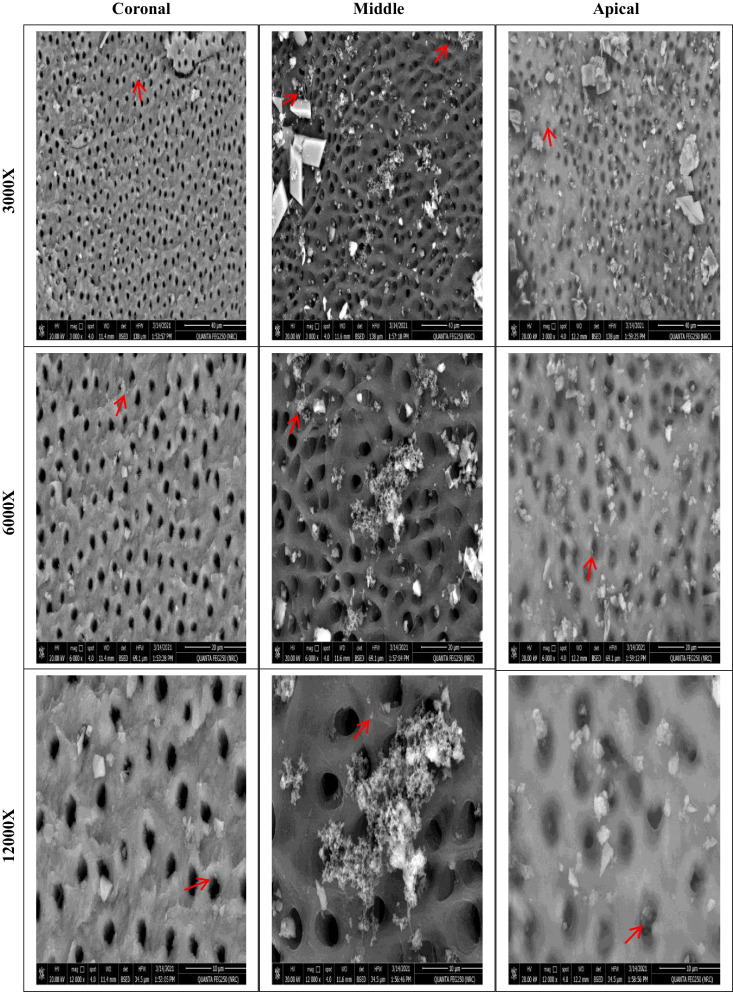
SEM photomicrographs at the three segments (coronal, middle, and apical) after preparation using XP-endo Shaper at different magnifications showing remaining bacteria and biofilm (red arrows)

**Fig. 5:**
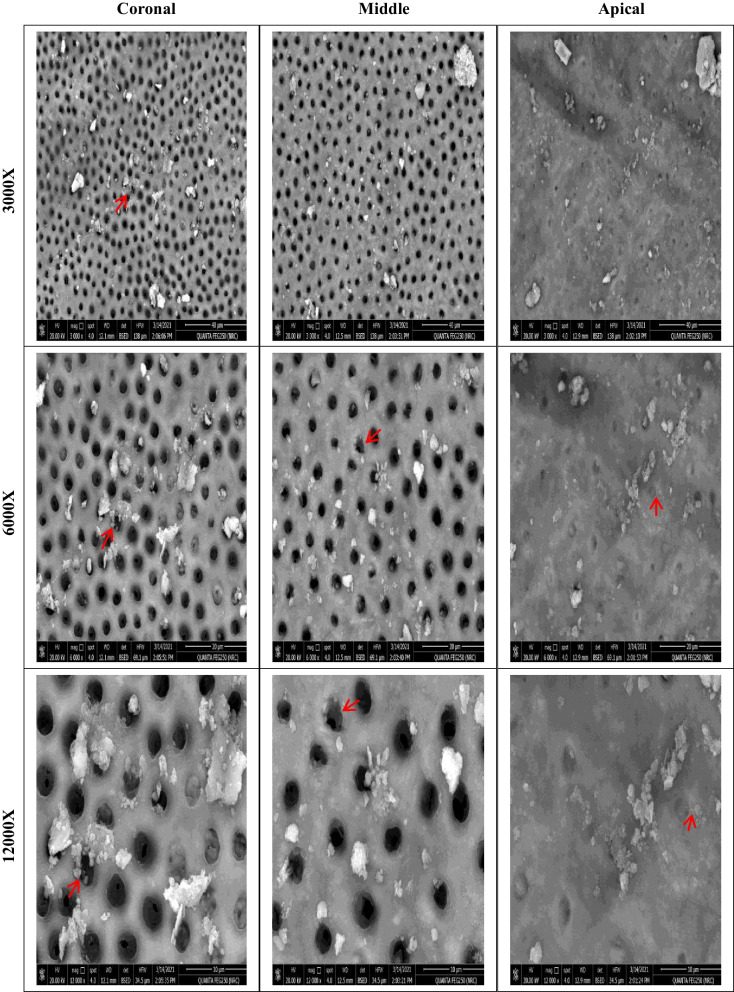
SEM photomicrographs at the three segments (coronal, middle, and apical) after preparation using Hyflex EDM at different magnifications showing remaining bacteria and biofilm (red arrows)

**Fig. 6:**
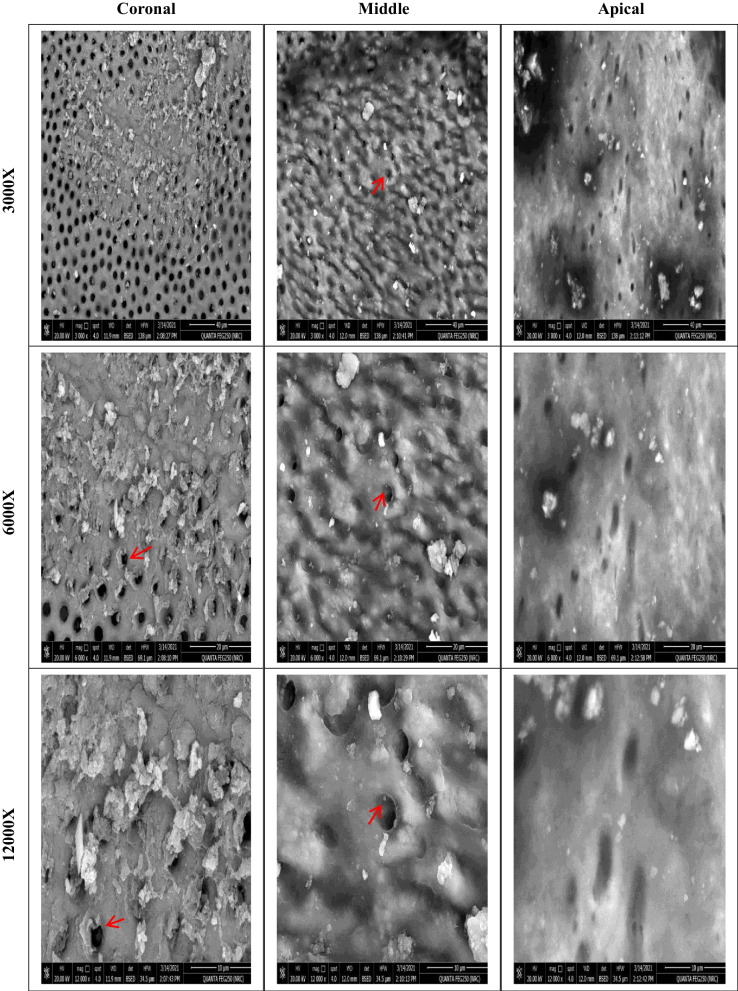
SEM photomicrographs at the three segments (coronal, middle, and apical) after preparation using One Curve at different magnifications showing remaining bacteria and biofilm (red arrows)

**Fig. 7:**
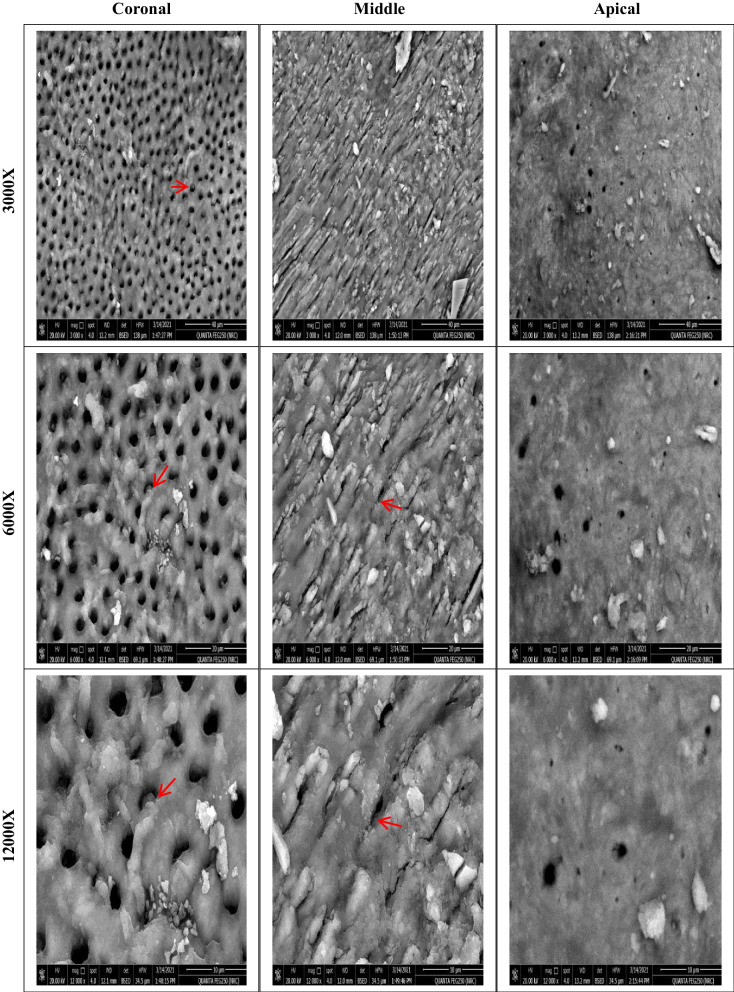
SEM photomicrographs at the three segments (coronal, middle, and apical) after preparation using Fanta. AFTM F One at different magnifications showing remaining bacteria and biofilm (red arrows)

Before instrumentation, the initial bacterial count analysis showed no significant differences among the four groups (*P* = .623). All files were significantly effective in reducing the mean bacterial count (*P* < .05). There was a significant difference in the number of bacterial CFU between files after instrumentation (*P* = .007). The lowest bacterial count (CFU) was observed with XPS, followed by HEDM and OC, with no significant difference between them. The highest bacterial count was observed with FO. SEM photomicrographs supported the CFU/mL, Fig. 3, where instrumentation disrupted the biofilm, Fig. 4, 5, 6, and 7.

## Discussion

Endodontic treatment mainly depends on eradication of bacteria and their produced toxins, followed by creating a 3-dimensional hermetic seal [1, 12]. This goal is challenging to obtain because of the complexity of root canal systems, where bacteria remain in anatomic irregularities of the root canal system, invading dentinal tubules of up to 500 µm and forming biofilms [28, 29]. *Enterococcus* *faecalis* was chosen because of its ability to form resistant biofilms in persistent apical pathosis [30, 31]. Our study has limitations that must be acknowledged. The mono-cultured biofilm model system was chosen to be simple in order to facilitate ease of preparation and maximize biofilm surface area for the exposure experiment [32].

Extracted human teeth were used rather than non-biological substrates, because human dentine is preferred as a substrate for *E. faecalis* biofilm growth to simulate the clinical performance. The protein portion of the dentin matrix initiated events in biofilm formation and irreversible bacterial adhesion to it [33].

Mandibular premolars with similar anatomy were selected to minimize morphological variations, and apical diameters not larger than the #15 K-file to standardize the microbial load, as single-file systems have limited shaping ability in wider canals [34].

Gamma radiation was used for sterilization because it does not include high temperature and pressure; therefore, the physical properties of dentin are not changed, and consequently do not negatively affect the adherence of *E. faecalis* [35].

Covering teeth apical foramina with epoxy resin was used to mimic a closed apical system in vivo preventing extrusion of debris and irrigant from the canal as apical vapor lock situation [36].

A pilot study proved the maturation age of biofilm after 3 weeks. Incubation times differed considerably, ranging from one to seventy days [21]. The effect of different disinfecting agents on biofilms was investigated, and it was discovered that after 3 weeks of growth, old biofilms increased in thickness, cell count and antimicrobial resistance with no significant difference for extra time. The majority of studies investigated the distribution of incubation time reveals that 21 days as the optimal incubation time [37, 38].

The teeth and irrigants were immersed in a warm water bath at 37 ± 1 °C to simulate body temperature in clinical conditions. At body temperature, the mechanical properties of Ni–Ti alloys can be affected mainly as XPS [25, 39, 40].

The previous in vitro studies have recommended using sodium hypochlorite as an irrigant to simulate clinical scenarios, so it was used in this study for all the experimental groups [23, 41].

In the present study, no significant difference was recorded in the mean bacterial count and consequently in the *E. faecalis* biofilm removal efficiencies of XPS, HEDM, and OC. This was in agreement with the results of Kaya et al. [23], who reported that instrumentation with HEDM and XPS resulted in significantly greater bacterial reduction. In addition, Pèrez et al. [42] and Amaral et al. [43] reported that XPS successfully reduced bacterial load in oval canals.

This may be attributed to the instruments included in this study being made from alloys with different manufacturing processes, geometries, and kinematics, where XPS from Max wire technology change the file from the martensite phase at body temperature to the austenite phase. This permits it to expand to size [#30/0.04] from original size giving the instrument a semi-circular shape that allows the file to perform eccentric rotary motion against the canal walls [44]. HEDM is manufactured using the electric discharge machining of a CM-wire, which makes the file extremely flexible and has high fracture resistance. This combination decreases the number of files needed for complete cleaning and shaping of the root canal without compromising preservation of the root canal configuration, which is consistent with the work of Devi et al. [45], who found that instrumentation with HEDM offered better cleaning efficacy than ProTaper Next and stainless-steel K-files, especially in the middle and apical segments. The OC is composed of a C-wire with a variable cross-section, which improves its performance [46].

HEDM and OC more effective than FO in terms of efficacy, It may be related to instrument design, metallurgical properties (alloy processing) and kinematics [47]. Hyflex EDM is manufactured from CM-Wire using innovative manufacturing process called electrical discharge machining (EDM). This process could harden the surface of file resulting in increased fracture resistance and superior cutting efficiency combined with flexibility of CM-Wire, so one file is required to clean the canal with preserving the anatomy with reduced transportation, ledging and perforation due to controlled memory properties [16]. One curve is manufactured by a unique manufacturing technique creating a controlled memory heat-treated NiTi called C-Wire which can be pre-bent for easier root canal preparation and removal of difficulties with increased blade flexibility and more fracture resistance for higher safety. It has a variable blade cross-section from triple-helical in apical 4 mm to S shape to enhance the centering and cutting ability in the apical third and removal of debris reaching middle and coronal parts [48]. The flat design of Fanta AF™ F One in one half of the cross section may result in the accumulation of more debris between the instrument and canal walls occupying the spaces created by the flat design instrument. This may lead to inconsistent cutting of the surrounding walls. Trapped debris also may reduce the instrument’s cutting surface that caused a less amount of dentin removal/second making it a time consuming instrument [49].

The current study reported that XPS is effective for bacterial reduction, which is in agreement with the results of Alves et al. [41], and Siddique et al. [50]. In addition, the results of our study coincide with those of Azim et al. [25], who found that XPS can touch more canal walls, leading to better disinfection. This result might be related to the classification of XPS as an adaptive core instrument with a small mass and expanding properties that allows targeting of the 3-dimensional of the canal while providing sufficient space for debris to escape [14].

Rodrigues et al. [51] reported that more bacteria could be eliminated with a larger apical preparation, because by enlarging the canal apically, more root canals could be touched by removing anatomical irregularities, adherent biofilms, and infected dentin. Therefore, XPS exhibited superior performance compared with the other files. On the other hand Matos Neto et al. [52] and Machado et al. [53] reported no significant differences between instruments of different sizes and tapers.

However, the results were inconsistent with those of Versiani et al. [54], who found that XPS had more unprepared root canal walls than i-race. This discrepancy can be explained by the different anatomy of the root canals of the teeth used in that study, where mandibular incisors were selected with long oval shaped canals. Also, this may be because of the comparison of the shaping abilities of a single-file system with those of multiple-file rotary systems, resulting in a longer irrigant contact time.

It has been expected to have greater bacterial remnants (CFU) in the cervical third than middle third followed by the apical third due to greater number of dentinal tubules in the corresponding portions of root canal system but in our study from SEM results, it was observed that there was less effective biofilm removal and maximum debris in the apical thirds of root canals which coincide with many studies [55].This might be attributed to complex root canal apical morphology and not enough apical preparation as several studies have demonstrated more efficacious irrigation in canals prepared to a greater taper [56, 57]. As irrigants did not adequately penetrate the apical third which has a narrower diameter than the middle and coronal thirds, however, the preparation size in all used systems permitted unimpeded 30-gauge needle penetration to 2 mm shorter than the working length [58].

To the best of our knowledge, no study has compared the efficacy of Fanta. AF™ F One and One Curve single-file systems for the reduction of bacterial load from root canals.

## Conclusion

Within the limitation of this study, the null hypothesis was partially accepted where single-files can satisfactorily clean and shape the root canals. XPS, HEDM, and OC were more effective than FO in eliminating *E. faecalis* biofilms from the infected root canals. However, none of these systems can eliminate biofilms completely. Further biofilm studies, is necessary to perform a comprehensive biofilm growth kinetics assay in order to identify and understand the biofilm maturation stage in different models.

## Data Availability

The datasets generated and analyzed during the current study are not publicly available due to (ownership of data) but are available from the corresponding author on reasonable request.
